# The Influence of Corporate Boundary Personnel Guanxi and Organizational Loyalty on Opportunistic Intentions – Based on Theory of Reasoned Action Model

**DOI:** 10.3389/fpsyg.2022.934012

**Published:** 2022-06-20

**Authors:** Shu-kuan Zhao, Jia-ming Cai

**Affiliations:** School of Business and Management, Jilin University, Changchun, China

**Keywords:** Guanxi, organizational loyalty, opportunism, dependence on the collaborator, boundary personnel

## Abstract

To understand the mechanism of boundary personnel opportunistic behaviors in collaborative R&D projects to reduce the risk of companies suffering from opportunism in collaboration. This study is conducted based on the context of collaborative R&D in the equipment manufacturing industry in Northeast China. This research mainly explored the mechanism of boundary personnel opportunistic intentions. Drawing on the theory of reasoned action (TRA), this study investigated the relationship between boundary personnel Guanxi, organizational loyalty, opportunistic attitudes, subjective norms, and intentions. In addition, this research examined the moderating role of the degree of dependence on the collaborator. In total, 524 valid questionnaires were finally collected. The data analysis results suggested that Guanxi inhibits opportunistic attitudes and subjective norms. Organizational loyalty promotes opportunistic attitudes and subjective norms. Opportunistic attitudes and subjective norms positively predict intentions. Opportunistic attitudes mediate between organizational loyalty and opportunistic intentions. Opportunistic subjective norms mediate between Guanxi and opportunistic intentions. Opportunistic subjective norms also mediate between organizational loyalty and opportunistic intentions. Dependence on the collaborator positively moderates the relationship between opportunistic attitudes and intentions. Therefore, it can be argued that in collaborative R&D in the equipment manufacturing industry, the corporate could stimulate boundary personnel to build good Guanxi to eliminate opportunism. At the same time, companies should lead employees to show loyalty properly, which opportunism is not wise in collaborative R&D. Finally, enterprises should objectively understand and evaluate the dependence relationship between the two partners in collaborative R&D to adopt the right strategy.

## Introduction

The equipment manufacturing industry is technology intensive. Collaborative R&D helps the industry grow rapidly (Xu et al., 2017). Theoretical research and practice showed that collaborative R&D among firms in the chain can improve business performance (Yang and Lin, 2020). The specific manifestation is to reduce cost and improve revenue (Um and Kim, 2018), improve market forecast accuracy through information exchange (Nimmy et al., 2019), strengthen communication between members within the industry (Zhang and Cao, 2018), and share resources among partners (Ramanathan and Gunasekaran, 2014). Equipment manufacturing is an important pillar industry in Northeast China (Liu et al., 2019). Led by many advanced enterprises, mature equipment manufacturing industry cluster has been formed in Northeast China with high-quality innovative resources (Cai et al., 2015). Relying on rich innovative resources, a lot of collaborative R&D activities upstream and downstream industry chains have been carried out in the industry (Wang, 2016). Although collaborative R&D is beneficial to industrial development and enterprises’ acquisition of competitive advantages, frequent opportunism seriously restricts the development of collaborative R&D and frustrates the enthusiasm of innovative subjects to participate in collaboration (Galvin et al., 2021).

Regarding research on inter-firm opportunism, scholars have mostly explored the governance mechanism of opportunism in the context of the purchaser–supplier relationships and joint ventures (Yang et al., 2017), with insufficient attention to opportunism in collaborative R&D (Yang et al., 2020). Collaborative R&D faces higher uncertainty, greater information asymmetry between collaborating parties, and potentially higher asset specialization, which is more likely to breed opportunism. Therefore, opportunism in the context of collaborative R&D cannot be ignored (Dickson et al., 2006). Existing research has identified several factors as antecedents of inter-firm opportunism (Jia et al., 2021). Based on these factors, scholars have explored the governance mechanisms of opportunism. Existing research generally classifies governance mechanisms for opportunism into formal and informal governance models (Luo, 2007). Luo (2007) believed that personal relationships will become more and more important as an informal governance mechanism. With the deepening of cooperation, the economic attributes of the transaction relationship between the two sides will continue to decrease while the social attributes will continue to rise. Some studies have further verified that the personal relationship between boundary personnel can inhibit the opportunism of cooperative enterprises to a certain extent (Wang et al., 2014; Shen et al., 2019). Furthermore, as the direct implementer of collaborative R&D projects, boundary personnel are engaged in representing enterprises, sharing information, managing conflicts, and solving problems, which play a key character in the development of cooperation and maintenance of the relationship between enterprises (Cai et al., 2021). Therefore, boundary personnel opportunism will harm collaborative R&D greatly. However, a few research studies are concentrating on boundary personnel opportunism. There is also no further study on the direct influence of the personal relationship between boundary personnel on their opportunism.

Chinese society used to regard an organization as an extension of the family and generalize the structure and operating principles of the family to organizations, which is a kind of pan-familism (Huangfu et al., 2013). Thus, the relationship between the junior to the senior in the family becomes the loyalty of the individual to the superior in the organization (Dingyu Jiang et al., 2003). This kind of organizational loyalty based on quasi-family is completely different from the Western concept of the social contract, which is a character concept in the Chinese context (Huangfu et al., 2013). Organizational loyalty is a kind of social connection between individuals and organizations, which exerts a subtle influence on individual psychological states and behavioral tendencies. This is also true for boundary personnel. Organizational loyalty, as a positive workplace emotion, is generally believed to promote positive workplace behaviors. A few studies have examined the relationship between organizational loyalty and negative workplace behaviors. This study examined the relationship between boundary personnel organizational loyalty and opportunism in collaborative R&D projects. The theory of reasoned action (TRA) assumes that an individual’s behavior is the result of rational decision making. The information input influences behavioral intentions through behavioral attitudes and subjective norms. Due to their special work role, boundary personnel establishes both horizontal Guanxi with the border personnel sent by the other company and bottom-up social relations with their own company. Both dimensions of social relationships serve as information inputs that influence individuals’ opportunistic attitudes and subjective norms, which in turn influence intentions. Guanxi between boundary personnel is a reflection of the horizontal social relationship. Organizational loyalty is a reflection of the bottom–up social relationship between the employee and the organization. This study further hypothesizes that Guanxi between boundary personnel from two parties will restrain the opportunistic intentions, while the organizational loyalty of boundary personnel to their organization will promote the opportunistic intentions. In addition, existing research has explored that inter-organizational dependence affects firm opportunism. This study further examined whether inter-firm dependence, as an environmental factor, has an impact on the boundary personnel opportunism rational decision-making process. This study was conducted on boundary personnel involved in collaborative R&D in the equipment manufacturing industry in Northeast China. Based on TRA, this study explored the effects of Guanxi and organizational loyalty on opportunistic attitudes and subjective norms. This study also tested the effects of opportunistic attitudes and subjective norms on intentions. In addition, this research investigated the moderating role of dependence on the collaborator.

## Literature Review and Hypotheses

### Opportunism in Business Cooperation

Opportunism in business cooperation refers to firms maximizing their self-interest at the sacrifice of collaborators (Williamson, 1985). Opportunism usually takes different forms, such as not taking responsibility, withholding relevant information, and not keeping their word (Galvin et al., 2021). The scholars of transaction cost economics suggested that opportunism occurs in situations of high uncertainty, high complexity, and the investment of large amounts of specialized assets that are difficult to change application (Moschandreas, 1997). Opportunism wastes innovation resources, reduces output, discourages collaborators from innovation, and constrains the long-term development of collaboration (Kang and Jindal, 2015).

Luo (2006) classified opportunism into two manifestations including strong and weak forms of opportunism. This classification has been adopted by many scholars (Zhao et al., 2021). The strong form of opportunism refers to “violation of the norms outlined in the express terms of the contract” and the weak opportunism refers to “violation of the tacit norms not expressively stated in the contract, but agreed upon in the cooperation.” Strong opportunism is more easily detected and thus more easily corrected by the cooperative system and may last for a shorter period. Weak opportunism, on the other hand, is less detectable and may last longer. Even if detected, there is no timely punishment for weak opportunism because they do not violate contractual norms (Luo et al., 2015). Thus, weak opportunism may have more lasting adverse effects on collaborative R&D projects (Luo et al., 2015). This study’s research objects are boundary personnel in collaborative R&D. Considering boundary personnel’s job characteristics and limits of authority, they are more likely to adopt weak form rather than strong form opportunism. Thus, this research concentrates on the weak form of opportunism, including (1) not giving full effort in cooperation but holding back; (2) violating knowledge sharing agreement; (3) hiding key resources needed by partners; (4) misrepresenting the strengths of the other party; and (5) deliberately obfuscating information or not revealing it in a timely manner it to the other party.

### Guanxi

Guanxi is a concept rooted in traditional Chinese Confucianism and has deeply influenced Chinese philosophy (Su et al., 2008; Liu et al., 2022). There are three principles of Guanxi operation. First of all, the basic principle is reciprocity (Luo et al., 2015). The principle requires both parties to give each other a “face” and return “favor” in the process of interaction. If one party does not give help to the other party in time of need and fulfill the obligation to return the favor, the Guanxi between the two parties will be damaged. Second, Guanxi is built on a long-term orientation. Chinese social norms require people to maintain harmonious and steady Guanxi (Luo, 2007). Third, Guanxi is not only an emotional expression but also a consideration based on utilitarianism. Both parties to Guanxi tend to obtain benefits from each other based on Guanxi, which is especially obvious in Chinese business activities (Park and Luo, 2001). Guanxi can smooth inter-firm collaboration (Luo et al., 2014). When scholars explored the informal governance of opportunism, the personal relationship attracted the attention of some scholars. Informal governance mechanisms such as Guanxi and formal governance mechanisms such as contract enforcement complement each other and reduce collaborating parties’ opportunism (Shen et al., 2020; Wang et al., 2021). Zhang et al. (2021) revealed that Guanxi between sales managers and salespersons inhibits the partner firm’s opportunism, and the deterrence effect is moderated by legal effectiveness, Confucianism, and organizational culture incongruence. Wang et al. (2014) displayed that managerial ties enhance inter-organizational trust, further improving the quality of supply chain information sharing and thus reducing supplier opportunism. Existing research has also focused on the impact of personal relationships between boundary personnel on collaborator opportunism. Shou et al. (2022) showed that buyer dependence increases supplier opportunism. But Guanxi between boundary personnel weakens the opportunism-promoting effects of buyer dependence. A previous study also showed that Guanxi between boundary personnel can reduce collaborator opportunism, in which partner asset specificity and legal enforceability can play moderation roles (Shen et al., 2020). Although existing studies have explored the inhibiting effect of Guanxi between boundary personnel on firm-level opportunism, neglected that Guanxi may also affect the individual-level opportunism. This research proposed that Guanxi may affect boundary personnel opportunism.

### Organizational Loyalty

In Chinese society, people and organizations have a bottom-to-top relationship that naturally reflects a sense of loyalty to the organization. Such a sense of loyalty is based on a mimetic family journey. What individuals show as filial piety in the family becomes loyalty in the organization (Huangfu et al., 2013). Therefore, the sense of loyalty is based on the proposed family, formed by a proposed kinship or blood relationship, which is fundamentally different from the concept of social contract developed in the Western culture based on the basic human rights of individual freedom and equality. However, with the development of the times and the progress of society, the Western concept of contract may also penetrate the Chinese concept of organizational loyalty, especially in modern corporate organizations. So that the concept of commitment based on contract also becomes one of the components of the Chinese concept of organizational loyalty (Huangfu et al., 2013). Therefore, from a review of empirical studies, Dingyu Jiang et al. (2003) identified similarities and differences between Chinese organizational loyalty and Western organizational commitment. They further defined Chinese organizational loyalty as “the process of familiarization, in which the individual’s role is closely aligned with the organization, and he or she is willing to put the organization’s interests above his or her own, and actively give to the organization” (Dingyu Jiang et al., 2003). Familiarization refers to the application of the coping styles learned by individuals in the family to the activities of the organization. The close integration of roles includes the emotional identification and internalization of the organization and the presentation of personal role responsibilities or obligations. Willingness to put the organization’s interests above one’s own refers to the individual’s willingness to comply with the organization or to sacrifice for the organization (Dingyu Jiang et al., 2003).

Some studies have explored the antecedent variables of organizational loyalty (Matzler and Renzl, 2006). Veloso et al. (2021) revealed that employee satisfaction contributes significantly to employee organizational loyalty and contextual performance. Nguyen (2021) found the factors affecting the employees’ loyalty in private joint-stock commercial banks in Mekong Delta. The result is that income, job characteristics, working environment, colleagues, and leadership are important factors. Cachn-Rodrguez et al. (2021) proposed that organizational legitimacy has a mediating effect between sustainability practices and employee loyalty. Other studies had estimated the influence of organizational loyalty on employees’ behaviors and intentions. Moynihan and Landuyt (2008) suggested that organizational loyalty can reduce employee turnover intention. However, existing research lacks attention to the negative consequences that organizational loyalty may trigger, such as pro-organizational unethical behavior.

### Theory of Reasoned Action

Ajzen and Fishbein (1977) proposed the TRA model. It explains the individual’s behavioral decision-making process based on the assumption that people are rational. People collect information to evaluate the meaning and consequences before committing a specific behavior. The theory model includes behavioral attitudes, behavioral subjective norms, behavioral intentions, and behaviors. Behavioral attitudes refer to an individual’s subjective attitudes toward adopting the behaviors, including the perception and evaluation of the behaviors and the assessment of the subsequent outcome of the behaviors. Behavioral subjective norms are an individual’s perceived evaluation by significant others of an individual taking a certain behavior. If those who are important to the individuals are in favor of (or against) the action, they are more likely (or less likely) to act. Behavioral intentions, on the other hand, refer to the willingness to engage in particular behaviors, which are prerequisites for actually performing the behaviors. Behavioral intentions are influenced by behavioral attitudes and subjective norms (Ajzen and Fishbein, 1977).

Existing studies have applied the TRA model to explore employee workplace behaviors. The TRA model can explain the mechanisms of workplace behaviors and predict the occurrence of behaviors. Casimir et al. (2012) applied the TRA model to explore the mechanisms of employee knowledge-sharing behavior. The study also extended the TRA model to validate the mediating role of IT usage between knowledge-sharing intention and knowledge-sharing behavior. Diethert et al. (2015) developed a model that integrated relevant self-directed and life-long learning motivation variables of earlier research into the general framework using TRA. The extended model has good predictive power. Existing studies have validated the excellent applicability of the TRA model to employee workplace behavior (Ng, 2020). This study applied the TRA model to explore the mechanism of the influence of Guanxi and organizational loyalty on boundary personnel opportunistic intentions.

### Dependence on the Collaborator

The organizational dependence of a firm is an important factor in determining whether opportunism by boundary personnel is more likely to occur (Provan and Skinner, 1989). Power dependency theory is mostly used to explore the balance of power and dependency relationships in dyadic business relationships (Jia and Yang, 2021). The power of one party is implicit in the dependence of the other party, so the power relationship is also the dependence relationship between the two parties. Dependence arises from resource advantages (Skowronski et al., 2022). A firm with resource advantages can influence the achievement of another party’s goal (Alrosjid et al., 2022). Power dependence theory suggests that unilateral dependence relationships breed more opportunism than interdependence relationships (Provan and Skinner, 1989; Casciaro and Piskorski, 2005). In a unilateral dependence situation, the dependent side can find substitutes more easily, which will be more likely to satisfy its interests by undermining the rights and interests of the relying party. Conversely, the relying party’s tendency to behave opportunistically will be inhibited by the high cost of finding a substitute (Provan and Skinner, 1989). It is difficult for both parties to find a substitute, and the conflict caused by opportunism can affect the mutual relationship and cause losses (Kumar et al., 1995). Therefore, both parties will refrain from opportunism. This research examines the moderating effects of unilateral dependence in collaborative R&D on boundary personnel opportunism.

### Hypotheses

#### Guanxi and the Theory of Reasoned Action Model

Qian et al. (2016) showed that boundary personnel Guanxi allows both partners to consciously restrain their behaviors to avoid harming each other and losing long-term benefits. Previous research revealed that in developing countries, boundary personnel’s Guanxi helps both partners to communicate and collaborate in a high-quality manner, to take responsibility, and solve problems together. Guanxi helps to reduce the possibility of conflict and to establish harmonious cooperation (Su et al., 2008). It can be observed that there is an effect of boundary personnel Guanxi on inter-firm relationships and inter-firm opportunism. However, the influence of Guanxi on boundary personnel’s opportunism has not been further explored in existing studies.

First, this study discusses the relationship between boundary personnel Guanxi and opportunistic attitudes. Boundary personnel represents their own companies in collaborative R&D. Their interests are tied to the interests of their companies. When boundary personnel commits opportunism against the other party, they not only hurt the other party but also hurt the interest of the boundary personnel from the other party. Guanxi operates based on a reciprocal, long-term orientation (Hwang, 1987). But opportunism goes against the guidelines of the Guanxi operation principle. Therefore, in the case of good Guanxi between two boundary personnel, one boundary personnel will perceive opportunism as contrary to the operational norms of Guanxi. Although opportunism may bring temporary benefits to the firm, boundary personnel will still inhibit their opportunism. Therefore, this research makes the following hypothesis.

H1: Guanxi between boundary personnel has a negative impact on opportunistic attitudes.

Second, this study tests the influence of Guanxi on opportunistic subjective norms. The code of Guanxi operation is the basic code of social interaction in China and is generally accepted by the public (Xiaotong, 1999). Chinese society is a word-of-mouth society of acquaintances. The word-of-mouth system oversees individuals’ adherence to the norms of Guanxi operation (Luo et al., 2016). When the Guanxi between boundary personnel is good, being opportunistic violates the Guanxi operation norms. Opportunism can be perceived as “treacherous,” “villainous,” and “short-sighted” by the important people, thus affecting the personal reputation system. This can further affect the individual’s access to social resources and even many aspects of work and life. Therefore, this research makes the hypothesis below.

H2: Guanxi between boundary personnel has a negative impact on opportunistic subjective norms.

#### Organizational Loyalty and the Theory of Reasoned Action Model

Guanxi between boundary personnel is a horizontal social association between individuals, and the organizational loyalty of the boundary personnel is a bottom–up vertical social association between an individual and the organization. Boundary personnel is embedded in both horizontal and vertical social relations, thus both dimensions of social relations have an impact on the boundary personnel opportunism.

This research argues that boundary personnel opportunism is essentially a form of pro-organizational unethical behavior. Pro-organizational unethical behavior is defined as altruistic rather than self-interested unethical behavior that is sometimes undertaken by employees for the benefit of the organization and its members (Umphress and Bingham, 2011). Boundary personnel acts opportunistically as an unethical behavior for their own company’s benefit in collaborative R&D, which meets the definition of pro-organizational unethical behavior. Although previous research has not explicitly stated that organizational loyalty positively affects employees’ pro-organizational unethical behavior, it has been shown that organizational commitment promotes pro-organizational unethical behavior (Fulmore and Fulmore, 2021). The concept of organizational loyalty, on the other hand, was developed with some of the connotations of the concept of organizational commitment. There is some overlap between the concepts of organizational commitment and organizational loyalty (Huangfu et al., 2013). Therefore, it is reasonable to extend the findings of Fulmore and Fulmore (2021). That is, in collaborative R&D, the boundary personnel with high organizational loyalty will closely combine his or her interests with the organization and maybe opportunism for both organizational and personal interests. Consequently, the boundary will have a strong subjective willingness to be opportunistic when conditions permit. In addition, considering the opportunistic subjective norms, when individuals are more loyal to the organization, they will generalize their “filial piety” to their “loyalty” to the organization. They will believe that being opportunistic to benefit the company is a manifestation of loyalty, which will be supported and encouraged by people around them. Based on the above derivation, this research proposes the below hypotheses.

H3: Boundary personnel’s organizational loyalty has a positive impact on opportunistic attitudes.

H4: Boundary personnel’s organizational loyalty has a positive impact on opportunistic subjective norms.

#### The Theory of Reasoned Action Model

Previous studies have shown that the TRA model can be used to explain employees’ workplace behaviors. For example, Ng (2020) showed that the TRA model has good applicability for explaining employees’ workplace knowledge-sharing behaviors. Moreover, Palm et al. (2020) showed that employees’ work-to-non-work integration behaviors can also be predicted by the TRA models, in which attitudes and subjective norms of work-to-non-work integration behaviors positively influence behavioral intentions. Nevertheless, the TRA model can be applied not only to positive workplace behaviors but also to predict work-related misbehaviors. For instance, Vardi and Weitz (2002) applied the TRA model to explain employee misbehavior, and their study showed that attitudes and subjective norms could influence behavioral intention. Opportunism, the subject of this study, is also essentially a form of workplace misbehavior. The application of TRA allows for the initial determination that opportunistic attitudes and subjective norms can positively influence intentions. Accordingly, the following hypotheses are proposed in this study.

H5: Boundary personnel’s opportunistic attitudes have a positive impact on opportunistic intentions.

H6: Boundary personnel’s opportunistic subjective norms have a positive impact on opportunistic intentions.

#### Mediating Effect

This research has preliminarily deduced that Guanxi negatively affects boundary personnel opportunistic attitudes. Opportunistic attitudes positively affect opportunistic intentions. It can be further deduced that opportunistic attitudes play a mediating effect between Guanxi and opportunistic intentions. In addition, the aforementioned deviation has also proposed that organizational loyalty positively influences opportunistic attitudes of boundary personnel. Opportunistic attitudes influence intentions. So it can be further inferred that opportunistic attitudes play a mediating effect between organizational loyalty and opportunistic intentions. Therefore, this study makes the following hypotheses.

H7: Opportunistic attitudes play a mediator between Guanxi and opportunistic intentions.

H8: Opportunistic attitudes play a mediator between organizational loyalty and opportunistic intentions.

It was also noted above that Guanxi would curb the opportunistic subjective norms. The opportunistic subjective norms would influence positively behavioral intention. Thus, this research further deduced that opportunistic subjective norms play a mediating effect between Guanxi and opportunistic intentions. Moreover, the aforementioned research has also deduced that organizational loyalty promotes opportunistic subjective norms. And opportunistic subjective norms influence intentions. Therefore, this study further deduced that opportunistic subjective norms play a mediating role between organizational loyalty and opportunistic intentions. This research proposes the following hypotheses.

H9: Opportunistic subjective norms play a mediator between Guanxi and opportunistic intentions.

H10: Opportunistic subjective norms play a mediator between organizational loyalty and opportunistic intentions.

#### Moderating Effect of Dependence on the Collaborator

When considering whether an individual adopts a certain behavior, the environment as a factor should be considered (Bandura, 1989). The dependency relationship between the collaborating firms is an important feature of the environment, which affects the behavior intentions of boundary personnel. Generally, one party depends on the other party because it has a comparative disadvantage over the other party due to the possession of technical and financial resources (Provan and Skinner, 1989). The more dependent a firm is on a collaborator in a transaction, the more costly it is to replace the collaborator, which makes them treasure the collaborator more. Thus, they are more disciplined in collaboration (Casciaro and Piskorski, 2005). The behavioral decision-making process of boundary personnel is influenced by inter-organizational dependency. Inter-organization dependency affects the opportunistic intentions of boundary personnel. When the boundary personnel enterprise is more dependent on the collaborator, the boundary personnel will restrain his or her opportunism. They try to avoid being opportunistic to hurt the collaborator. In particular, when dependence on the collaborator is high, the facilitation effect of opportunistic attitudes on opportunistic intentions is inhibited. For the same reason, dependence on the collaborator also negatively moderates the facilitation effect of opportunistic subjective norms on opportunistic intentions. This research proposes the following hypotheses.

H11: The degree of dependence on the collaborator negatively moderates the relationship between opportunistic attitudes and intentions.

H12: The degree of dependence on the collaborator negatively moderates the relationship between opportunistic subjective norms and intentions.

**FIGURE 1:**
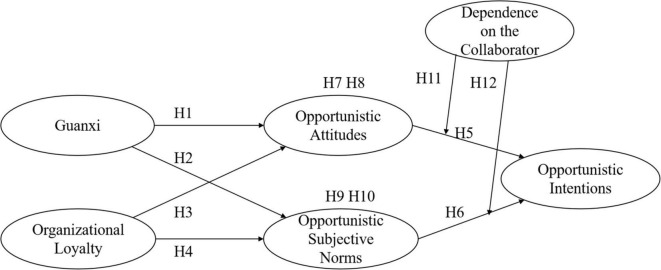
Theoretical framework.

Figure 1 is the research model.

## Research Method

### Subjects and Sampling Process

The study contacted a total of 30 executives in the equipment manufacturing industry in Northeast China. In total, 21 of them explicitly expressed their willingness to participate in the research project. They provided the list of boundary personnel, as well as their main job responsibilities. The team of this study finally selected 1,102 boundary personnel and distributed questionnaires to them. To ensure the efficiency of the questionnaire collection, this study used the professional questionnaire platform “Questionnaire Star.” The research team distributed the questionnaires from 1 January to 31 January 2022. Questionnaires with short answer times, consecutive questions with the same answers, or not passing the attention test were identified as invalid questionnaires. Finally, 524 valid questionnaires were received, with a valid rate of 47.55%.

### Measurement

This questionnaire investigated the basic information of boundary personnel and the research model variables. The basic information of boundary personnel includes the education experience background, gender, enterprise ownership, job position, and working years. The research model variables include Guanxi between boundary personnel, organizational loyalty, opportunistic attitudes, opportunistic subjective norms, opportunistic intentions, and dependence on the collaborator.

The measurement scales for the latent variables were all derived from well-established scales from existing studies. Regarding the measurement of Guanxi between boundary personnel, this study mainly referred to the studies of Lee and Dawes (2005); Zhuang et al. (2010), Huang et al. (2011), and Zhang and Zhang (2013). Six items were selected and adapted to fit the collaborative R&D context. The measurement of organizational loyalty came from Dingyu Jiang et al. (2003). The final measurement scale includes five items. This research referred to Ng (2020) and Luo (2006) to design scales to measure opportunistic attitudes, opportunistic subjective norms, and opportunistic intentions. The measurement of dependence on the collaborator is based on Lusch and Brown (1996) including four items. Because this study was conducted in China, the original measurements were translated from English to Chinese by professional translators. To check the accuracy of the translation, they were then back-translated into English by two other professional translators for comparison with the original scale. The questionnaire used a Likert seven-point scale, from 1 representing strongly disagree to 7 representing strongly agree.

### Method

In this study, the partial least squares (PLS) method was used to test the theoretical hypotheses. Different from the covariance-based structural equation model (CB-SEM), PLS-SEM uses a component-based analysis. It is generally agreed that PLS-SEM can maintain robust results despite small sample sizes or deviations from normal measurement data, and can achieve maximum prediction results (Lohmoller, 1988). The analysis software used in this study was smart PLS 3.0.

## Results

### Descriptive Statistical Analysis

A total of 524 valid questionnaires were finally collected. The background information of the subjects was investigated in this study. In total, 60.7% of the subjects were men and 88.2% of the subjects graduated from college or university. The subjects working for 6–9 years were the most, accounting for 60.5%. In total, 63.2% of the subjects were from private companies and 50.4% of the subjects were middle-level employees. Table 1 shows the frequency distribution.

**TABLE 1:** Frequency distribution.

Variable	Label	Frequency	Percent
Gender	Male	318	60.7
	Female	206	39.3
Educational	High school or below	3	0.6
	College/University	462	88.2
	Master or above	59	11.3
Working years	Under 2 years	11	2.1
	3–5 years	153	29.2
	6–9 years	317	60.5
	≥10 years	43	8.2
Enterprise type	State-owned enterprises	85	16.2
	Private enterprises	331	63.2
	Joint ventures and foreign companies	97	18.5
	Else	11	2.1
Position	High-level	30	5.7
	Middle-level	264	50.4
	Basic-level	230	43.9

Table 2 shows the statistical analysis of each construct. The construct with the highest mean is organizational loyalty. The construct with the lowest mean is opportunistic intentions. The construct with the highest standard deviation is opportunistic subjective norms. The construct with the lowest standard deviation is opportunistic attitudes. Based on the values of skewness and kurtosis, it can be inferred that the data conform to a normal distribution.

**TABLE 2:** Descriptive statistical analysis.

	Mean	Standard deviations	Skewness	Kurtosis
GX	3.765	1.454	0.737	–0.059
OL	5.092	1.369	–1.512	2.102
OA	3.668	1.290	0.153	–0.464
OSN	4.337	1.486	–0.783	–0.027
OI	3.160	1.437	0.348	–0.643

*GX, Guanxi; OL, organizational loyalty; OA, opportunistic attitudes; OSN, opportunistic subjective norms; OI, opportunistic intentions; DC, dependence on the collaborator.*

### Measurement Model Verification

Existing studies recommended that each item factor loading should be greater than 0.7, composite reliability (CR) and Cronbach’s α of each construct should be higher than 0.7, and the average variance extracted (AVE) should be larger than 0.5 (Fornell and Larcker, 1981; Nunnally and Bernstein, 1994). As shown in Table 3, the results of our empirical data suggested that the convergent validity of the constructs is reasonable.

**TABLE 3:** Convergent validity.

Construct	Factor loading	Cronbach’s α	CR	AVE
GX	0.799–0.867	0.915	0.934	0.702
OL	0.806–0.892	0.929	0.944	0.739
OA	0.787–0.904	0.917	0.938	0.752
OSN	0.842–0.899	0.915	0.936	0.746
OI	0.838–0.881	0.930	0.944	0.739

This research adopted the AVE method to verify the discriminant validity. Fornell and Larcker (1981) suggested that the AVE square root of a dimension (the bold figures in Table 4) is greater than the Pearson’s correlative coefficients with another dimension (the figures under the diagonal in Table 4), indicating the dimension is discriminant with another dimension. The data show that the constructs in this study have discriminant validity.

**TABLE 4:** Discriminant validity.

	GX	OL	OA	OSN	OI
GX	**0.838**				
OL	−0.421	**0.860**			
OA	−0.346	0.463	**0.867**		
OSN	−0.571	0.342	0.599	**0.864**	
OI	−0.361	0.134	0.375	0.540	**0.860**

*The bold value of the diagonal is the square root of AVE.*

### Structural Model Result

#### Path Analysis

The goodness-of-fit (GOF) indices were used to estimate the model fit of the proposed model. The calculation formula is $\text{GOF}=\sqrt{\bar{\text{AVE}}\times \bar{R^{2}}}$. In general, the larger the GOF, the better the fitness of the model is. The GOF of less than 0.1 is considered weak fitness. The GOF between 0.1 and 0.25 is regarded as low fitness. The GOF between 0.25 and 0.36 is deemed to be fair fitness. And the GOF above 0.36 is considered high fitness (Vinzi et al., 2010). The GOF of this study was 0.464, indicating that this study model has high fitness.

**FIGURE 2:**
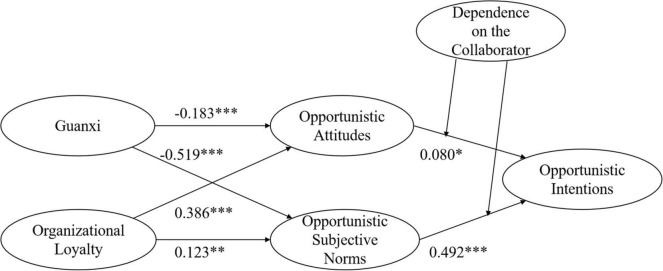
Structural model, *p < 0.05, ^**^p < 0.01, ^***^p < 0.001.

The results of the path analysis between the variables are shown in Table 5. Figure 2 shows the statistical model results.

**TABLE 5:** Path analysis.

	Path coefficient	Standard deviation	*T*-value	*P*-value	*R* ^2^	*Q* ^2^
GX → OA	–0.183	0.052	3.500	0.000	0.242	0.180
OL → OA	0.386	0.051	7.519	0.000		
GX → OSN	–0.519	0.046	11.161	0.000	0.338	0.251
OL → OSN	0.123	0.044	2.818	0.005		
OA → OI	0.080	0.039	2.076	0.038	0.296	0.214
OSN → OI	0.492	0.043	11.516	0.000		

(1) The standardized coefficient of Guanxi to opportunistic attitudes is −0.183 (*T* value = 3.500, *p* = 0.000 < 0.001), supporting H1. The findings suggest that Guanxi negatively and significantly influences opportunistic attitudes.
(2) The standardized coefficient of Guanxi to opportunistic subjective norms is −0.519 (*T* value = 11.163, *p* = 0.000 < 0.001), supporting H2. The results confirm that Guanxi negatively and significantly influences opportunistic subjective norms.
(3) The standardized coefficient of organizational loyalty to opportunistic attitudes is 0.386 (*T* value = 7.519, *p* = 0.000 < 0.001), supporting H3. This suggests that the effect of organizational loyalty on opportunistic attitudes is significant.
(4) The standardized coefficient of organizational loyalty to opportunistic subjective norms is 0.123 (*T* value = 2.818, *p* = 0.005 < 0.01), supporting H4. This indicates that organizational loyalty positively and significantly influences opportunistic subjective norms.
(5) The standardized coefficient of opportunistic attitudes to opportunistic intentions is 0.080 (*T* value = 2.076, *p* = 0.038 < 0.05), supporting H5. This demonstrates that opportunistic attitudes can significantly influence opportunistic intentions.
(6) The standardized coefficient of the subjective norms to the intention of opportunistic is 0.492 (*T* value = 11.516, *p* = 0.000 < 0.001), supporting H6. This reveals that subjective norms of opportunism can significantly influence opportunistic intentions.

*R*^2^ indicates the explanatory power of the exogenous variable. The *R*^2^ > 0.67 for the latent variable indicates a high explanatory power for this variable. The *R*^2^ > 0.33 for the latent variable indicates fair explanatory power for the variable. The *R*^2^ > 0.19 for the latent variable indicates a low explanatory power for this variable (Chin, 1998). The *R*^2^ value for opportunistic attitudes is 0.242. The *R*^2^ value for opportunistic subjective norms is 0.338. The *R*^2^ value for opportunistic intentions is 0.296. All of the *R*^2^ values indicate a reasonable explanatory power. The values of Q^2^ for opportunistic attitudes (*Q*^2^ = 0.180), opportunistic subjective norms (*Q*^2^ = 0.251), and opportunistic intentions (*Q*^2^ = 0.214) are greater than zero, indicating that the proposed model has sufficient predictive power.

#### Indirect Effect Analysis

The results of the tests for indirect effects are reported in Table 6. Guanxi has an impact on opportunistic intentions through opportunistic attitudes (*T* = 1.443 < 1.96, *p* = 0.149 > 0.05), indicating that the indirect effect does not exist and does not support H7. The testing results of organizational loyalty influencing opportunistic intentions through opportunistic attitudes are *T* = 2.213 > 1.96, *p* = 0.027 < 0.05, indicating that the indirect effect exists and that H8 is valid. Guanxi influences opportunistic intentions through opportunistic subjective norms (*T* = 6.900 > 1.96 and *p* = 0.000 < 0.001), indicating that the indirect effect persists and H9 is valid. The testing results of organizational loyalty influencing opportunistic intentions through opportunistic subjective norms are *T* = 2.880 > 1.96, *p* = 0.004 < 0.01, indicating that the indirect effect exists significantly (H10 is supported).

**TABLE 6:** Results of the indirect effect.

	Indirect effect	Standard deviation	*T*-value	*P*-values
GX – > OA – > OI	–0.015	0.010	1.443	0.149
OL – > OA – > OI	0.031	0.014	2.213	0.027
GX – > OSN – > OI	–0.255	0.037	6.900	0.000
OL – > OSN – >OI	0.061	0.021	2.880	0.004

#### Moderation Effect Analysis

**FIGURE 3:**
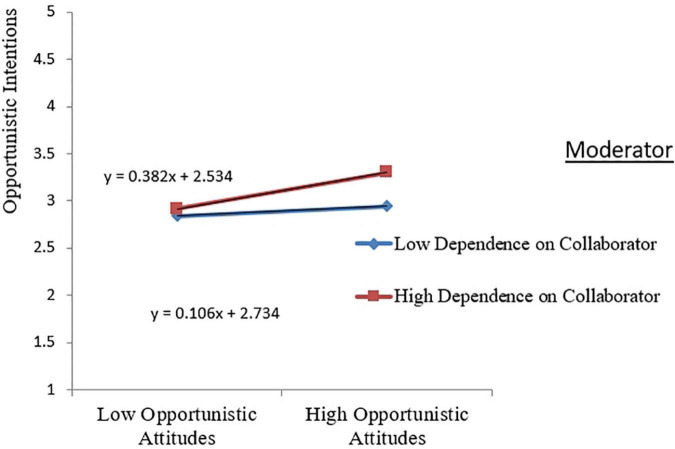
Moderating effect of dependence on the collaborator.

Table 7 shows the results of the moderation effect analysis. The effect of opportunistic attitudes×dependence on the collaborator on opportunistic intentions is 0.069 (*T* = 2.125 > 1.96, *p* = 0.034 < 0.05), indicating the presence of a moderating effect of dependence on the collaborator. Figure 3 is a diagram of this moderating effect. The results suggest that although there is a moderating effect of dependence on the collaborator, it is contrary to H11. This study hypothesized that there would be a negative moderating effect of dependence on the collaborator. However, the result shows that there is a positive moderating effect. Furthermore, the effect of the opportunistic subjective norms×dependence on the collaborator on the opportunistic intentions is 0.013 (*T* = 0.364 < 1.96, *p* = 0.716 > 0.05), indicating that this moderating effect does not exist, and H12 is not valid.

**TABLE 7:** Results of the moderation effect.

	Original sample	Standard deviation	*T*-value	*P*-values
OA × DC- > OI	0.069	0.033	2.125	0.034
OSN × DC- > OI	0.013	0.037	0.364	0.716

## Discussion

This study investigated boundary personnel Guanxi and organizational loyalty on the opportunistic intentions in the context of collaborative R&D in the equipment manufacturing industry in Northeast China. This study applied the TRA model to test the influence of boundary personnel Guanxi and organizational loyalty on opportunistic attitudes, opportunistic subjective norms, and opportunistic intentions. The mediating roles of opportunistic attitudes and opportunistic subjective norms were also investigated, as well as the moderating role of dependence on the collaborator.

### Theoretical Contributions

Boundary personnel who represent the enterprise and carry out collaborative R&D activities with partners are the direct executors of joint innovation activities (Mäkelä et al., 2019). Boundary personnel establishes social ties with boundary personnel from the collaborator and their organization (Aldrich and Herker, 1977). They form Guanxi between boundary personnel and organizational loyalty to their party. This study found that the level of Guanxi between boundary personnel in the equipment manufacturing industry collaborative R&D in Northeast China was not high. Still, the level of boundary personnel’s organizational loyalty to their organization was high. Among them, the inhibitory effect of Guanxi on opportunism was consistent with existing studies. It has been shown that personal relationships promote compliance with relationship norms (Zhou et al., 2015), and discourage opportunism of the partners (Luo, 2007; Zhou et al., 2015; Huang et al., 2016; Lin and Ho, 2021). The present study further suggests that Guanxi can have a disincentive effect on both attitudes and subjective norms of opportunism. This suggests that Guanxi affects both individual’s subjective assessment of opportunism and an individual’s perceived important people’s opportunism opinions. When Guanxi between boundary personnel is good, individuals subjectively perceive that opportunism will have adverse effects. Boundary personnel will also believe that social expectations discourage them from opportunism.

Previous research has shown that organizational commitment may trigger pro-organizational unethical behavior of individuals in some situations (Grabowski et al., 2019; Fulmore and Fulmore, 2021). Because the construct of organizational loyalty absorbed the connotation of organizational commitment at the beginning of its development, this study infers that the organizational loyalty of boundary personnel triggers opportunism in collaborative R&D (i.e., pursuing the interests of the host firm at the expense of the partner). The results of the present study, in a preliminary way, confirmed the plausibility of this inference. It also validated and extended the conclusions of Fulmore and Fulmore (2021) once again. The results indicate that organizational loyalty positively influences opportunistic attitudes and subjective norms. The interests of boundary personnel are highly aligned with those of the organization, so they will perceive opportunism as beneficial to both themselves and the organization. So boundary personnel with high organizational loyalty may also have high opportunistic attitudes. Besides, employees with high levels of organizational loyalty may also be more susceptible to the influence of their surroundings, such as pressure from leaders, co-workers, friends, and relatives. Opportunism is consistent with the interests of the firm in the short run. So boundary personnel with high levels of organizational loyalty will perceive that important people around them want them to behave opportunistically in collaborative R&D to show their loyalty to the firm. This result is also in line with the operating principles of Chinese word-of-mouth and acquaintance societies. Individuals place a high value on whether their behavior meets social expectations (Xiaotong, 1999).

In studies of workplace behaviors, many results revealed that attitudes and subjective norms could predict behavioral intentions (Mishra et al., 2014; Ng, 2020). Mishra et al. (2014) concluded that workplace green information technology acceptance attitude and subjective norms could predict behavioral intention. TRA also has good applicability to workplace unethical behavior (Lin et al., 2018). Vardi and Weitz (2002) indicated that workplace misbehaviors can also be predicted by TRA. The opportunism of boundary personnel is essentially workplace misconduct. This study’s results demonstrated that opportunistic attitudes and subjective norms have positive effects on intentions. This finding is consistent with previous research.

This study examined the mediating roles of opportunistic attitudes and subjective norms between Guanxi, organizational loyalty, and opportunistic intentions. The results show that opportunistic attitudes do not mediate between Guanxi and opportunistic intentions. But opportunistic subjective norms mediate between Guanxi and opportunistic intentions. The results also reveal that opportunistic attitudes and subjective norms mediate between organizational loyalty and opportunistic intentions. Guanxi can inhibit opportunistic intentions by influencing subjective norms. On the other hand, organizational loyalty promotes opportunistic intentions through opportunistic attitudes and subjective norms. Therefore, organizational loyalty may play a more critical role than Guanxi in rational decision making for the opportunism of boundary personnel.

Given the existence of dependency asymmetry among most collaborative R&D collaborators, this study further explores the moderating role of dependence on the collaborator in the boundary personnel opportunism decisions. Previous studies have primarily argued that dependence on the partner inhibits one’s opportunism (Provan and Skinner, 1989; Handley et al., 2019). However, this study concluded that dependence on the cooperating party positively moderates the relationship between boundary personnel opportunistic attitudes and intentions. When boundary personnel believes that their side is highly dependent on the partner, their opportunistic intention will be higher when their attitude is certain. The reason may be that organizational dependence arises from the resource asymmetry between the two parties, and the more dependent on the partner, the greater the resource advantage occupied by the partner. When boundary personnel assesses that opportunism is warranted, there may be a greater incentive to obtain scarce resources from the partner through opportunism. Moreover, the form of the opportunism explored in this study is weak. Weak opportunism is not easily detected by the partner. Even if detected, it is not easily punished. So boundary personnel does not fear retaliation from the partner for their opportunism.

#### Practice Implications

In collaborative R&D projects, boundary personnel is the ultimate direct executors of the collaboration, and opportunism will seriously negatively impact the collaborative R&D project (Zhang et al., 2019). A weak form of opportunism, in particular, is difficult to detect, more concealed, and produces more far-reaching adverse effects (Luo, 2006). The opportunism of boundary personnel not only affects the output of collaborative R&D projects but also affects the reputation of the company and makes it difficult to find new partners (Zhang et al., 2019). The results of this study suggest that Guanxi between boundary personnel inhibits attitudes and subjective norms toward opportunism, which further hinder intentions. Therefore, the development of appropriate Guanxi among boundary personnel should be encouraged.

Organizational loyalty is an employee’s positive emotion toward the organization (Huangfu et al., 2013) and can be effective in enhancing positive workplace behaviors such as the cooperative intentions of knowledge workers (Lifa et al., 2014). However, this study shows that organizational loyalty would positively influence boundary personnel opportunistic intentions. Employees with high levels of organizational loyalty may tend to believe that their leaders and colleagues expect them to behave opportunistically. This reminds managers that employees should be properly guided to adapt their misconceptions about opportunism. Managers should inform employees that opportunism in collaborative R&D projects is not a wise choice to show loyalty to the organization. Opportunism is contrary to the long-term interests of the organization. The organization discourages opportunism by any boundary personnel in collaborative R&D projects.

In addition, companies should reasonably judge the resources occupied by both partners in collaborative R&D, as well as the power–dependency relationship arising from resource asymmetry. Companies should take the right cooperation strategy based on the level of interdependence of both parties. This includes the training and management of boundary personnel. Companies should require boundary personnel to adopt appropriate working methods in collaborative R&D projects based on the dependencies of both parties.

### Limitations and Future Research Directions

First, this study was conducted in the equipment manufacturing industry in Northeast China, and the industry context has certain limitations. This research can be repeated in other industries in the future to improve the scope of application. Second, China is a vast country, and the economic development of different regions varies greatly. This study was conducted in the less economically developed northeast region of China, so the results of this study may not be applicable in other regions. Finally, opportunism is unethical, and subjects may have social legitimacy concerns when responding to the behavior. Although collected anonymously in this study, there would still likely be systematic social legitimacy issues.

Future research can be conducted in the following directions. First, studies can be conducted in different industries and geographic regions of China to compare the variability of the effects of Guanxi and organizational loyalty on the opportunism of boundary personnel across regions and industries. Second, this study has tentatively concluded that Guanxi among boundary personnel can deter their opportunism. Future research should further explore how to develop Guanxi among boundary personnel to better exploit the deterrent effect of Guanxi. Again, organizational loyalty as a positive emotion has been considered an antecedent variable for positive employee behavior. This study found that organizational loyalty may induce unethical behavior. Future research should further explore the boundaries of the double-edged sword effect of organizational loyalty. Finally, this study explored the moderating effect of inter-organizational dependencies on rational decision making of opportunistic behaviors of boundary personnel. The level of moderating variables can be enriched in the future. For example, the moderating role of the micro-environment of the boundary personnel’s organization could be considered, such as organizational culture and organizational structure. The influence of the macro social environment could be included as well, such as the social legal system and the social influence of Confucianism.

## Data Availability Statement

The raw data supporting the conclusions of this article will be made available by the authors, without undue reservation.

## Ethics Statement

Ethical review and approval was not required for the study on human participants in accordance with the local legislation and institutional requirements. Written informed consent from the patients/participants legal guardian/next of kin was not required to participate in this study in accordance with the National Legislation and the Institutional Requirements.

## Author Contributions

S-KZ and J-MC: conceptualization, formal analysis, investigation, methodology, validation, and writing – original draft, review, and editing. Both authors contributed to the article and approved the submitted version.

## Conflict of Interest

The authors declare that the research was conducted in the absence of any commercial or financial relationships that could be construed as a potential conflict of interest.

## Publisher’s Note

All claims expressed in this article are solely those of the authors and do not necessarily represent those of their affiliated organizations, or those of the publisher, the editors and the reviewers. Any product that may be evaluated in this article, or claim that may be made by its manufacturer, is not guaranteed or endorsed by the publisher.
